# *cis*-Dichloridobis(*N*,*N*,*N*′,*N*′-tetra­methyl­ethane-1,2-diamine)­platinum(II)

**DOI:** 10.1107/S1600536812048295

**Published:** 2012-11-30

**Authors:** Abdullah M. Asiri, Muhammad Nadeem Arshad, Muhammad Ishaq, Khalid A. Alamry, Tanveer Hussain Bokhari

**Affiliations:** a Chemistry Department, Faculty of Science, King Abdulaziz University, PO Box 80203, Jeddah 21589, Saudi Arabia; b Center of Excellence for Advanced Materials Research (CEAMR), Faculty of Science, King Abdulaziz University, PO Box 80203, Jeddah 21589, Saudi Arabia; c Department of Chemistry, Government College University, Faisalabad 38000, Pakistan

## Abstract

In the title complex, [PtCl_2_(C_6_H_16_N_2_)], the Pt^II^ atom adopts a distorted *cis*-PtN_2_Cl_2_ square-planar coordination geometry. The five-membered chelate ring adopts a twisted conformation. In the crystal, weak C—H⋯Cl hydrogen bonds link the mol­ecules into (001) sheets.

## Related literature

For related structures, see: Abellán-López *et al.* (2012 ▶); Boyle *et al.* (2004 ▶).
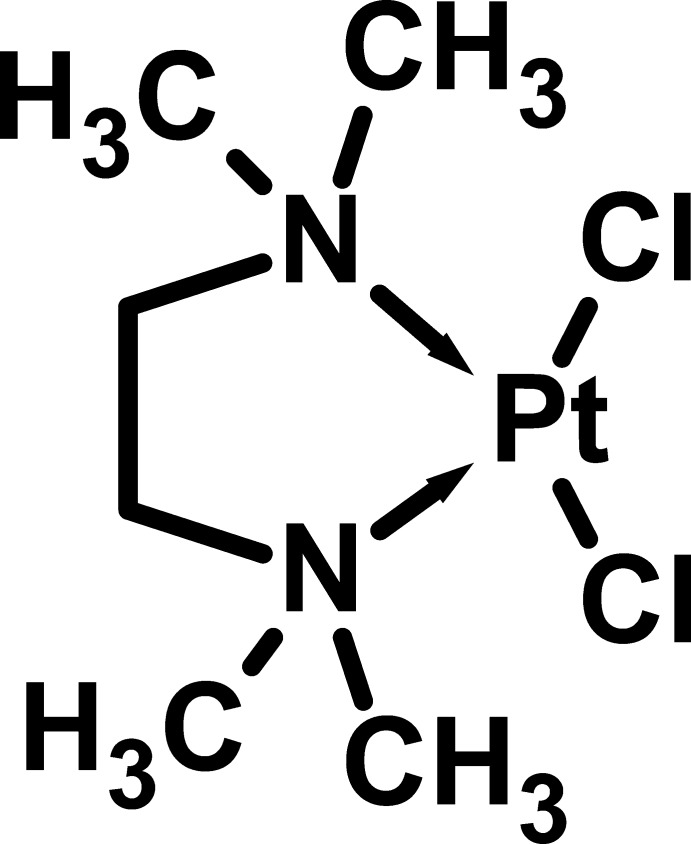


## Experimental

### 

#### Crystal data

[PtCl_2_(C_6_H_16_N_2_)]
*M* *_r_* = 382.20
Monoclinic, 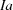

*a* = 11.8893 (2) Å
*b* = 6.0207 (1) Å
*c* = 15.8036 (3) Å
β = 110.549 (2)°
*V* = 1059.27 (3) Å^3^
*Z* = 4
Cu *K*α radiation
μ = 28.99 mm^−1^
*T* = 296 K
0.22 × 0.21 × 0.07 mm

#### Data collection

Agilent SuperNova (Dual, Cu at zero, Atlas, CCD) diffractometer
Absorption correction: multi-scan (*CrysAlis PRO*; Agilent, 2012 ▶) *T*_min_ = 0.149, *T*_max_ = 1.000
6899 measured reflections
2091 independent reflections
2067 reflections with *I* > 2σ(*I*)
*R*_int_ = 0.047

#### Refinement

*R*[*F* ^2^ > 2σ(*F* ^2^)] = 0.044
*wR*(*F* ^2^) = 0.116
*S* = 1.03
2091 reflections
105 parameters
2 restraints
H-atom parameters constrained
Δρ_max_ = 1.83 e Å^−3^
Δρ_min_ = −2.02 e Å^−3^
Absolute structure: Flack (1983 ▶), 1005 Friedel pairs
Flack parameter: −0.02 (2)

### 

Data collection: *CrysAlis PRO* (Agilent, 2012 ▶); cell refinement: *CrysAlis PRO*; data reduction: *CrysAlis PRO*; program(s) used to solve structure: *SHELXS97* (Sheldrick, 2008 ▶); program(s) used to refine structure: *SHELXL97* (Sheldrick, 2008 ▶); molecular graphics: *PLATON* (Spek, 2009 ▶); software used to prepare material for publication: *WinGX* (Farrugia, 2012 ▶) and *X-SEED* (Barbour, 2001 ▶).

## Supplementary Material

Crystal structure: contains datablock(s) I, global. DOI: 10.1107/S1600536812048295/hb7001sup1.cif

Structure factors: contains datablock(s) I. DOI: 10.1107/S1600536812048295/hb7001Isup2.hkl

Additional supplementary materials:  crystallographic information; 3D view; checkCIF report

## Figures and Tables

**Table 1:** Selected bond lengths (Å)

Pt1—N1	2.071 (7)
Pt1—N2	2.076 (6)
Pt1—Cl1	2.292 (4)
Pt1—Cl2	2.304 (5)

**Table 2:** Hydrogen-bond geometry (Å, °)

*D*—H⋯*A*	*D*—H	H⋯*A*	*D*⋯*A*	*D*—H⋯*A*
C1—H1*B*⋯Cl1^i^	0.96	2.79	3.724 (8)	166
C4—H4*A*⋯Cl1^ii^	0.97	2.82	3.596 (7)	137

Symmetry codes: (i) 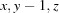; (ii) 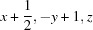.

**Fig. 1.**
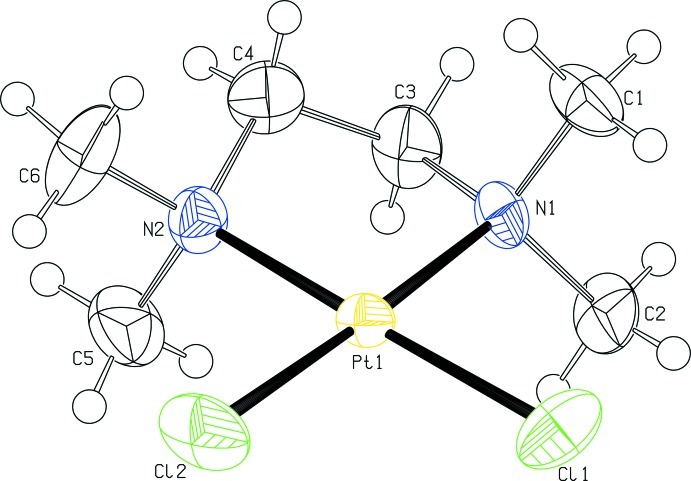
The molecular structure of title complex with 50% probability thermal ellipsoids.

**Fig. 2.**
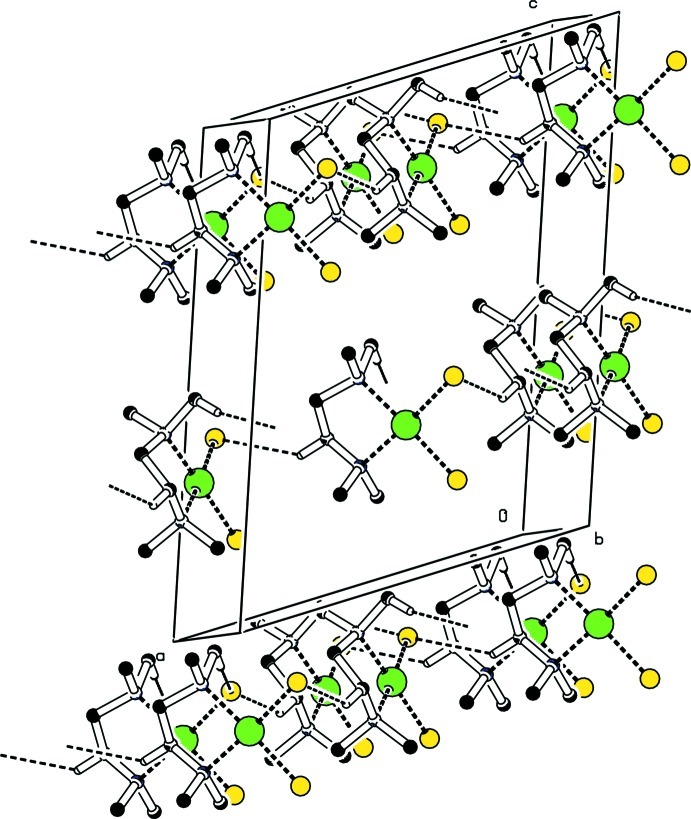
The packing diagram showing hydrogen bonds, drawn using dashed lines. Hydrogen atoms not involved in bonding have been omitted for clarity.

## supplementary crystallographic information

### Comment

The title compound is structurally related with already reported structures of complexes of the type of [MX2(N–N)] (N–N = chelating nitrongen donor ligand) like *cis*-diiodido(*N*,*N*,*N*',*N*' -tetramethylethylenediamine-*k*^2^N,N')palladium(II) (Abellán-López, *et al.* 2012) (II) and cis-dichloro(*N*,*N*,*N*'*N*'-tetramethyl-1,2-diaminoethane) palladium(II) (Boyle *et al.* 2004) (III).

In the structure of molecule I shown in Fig. 1, geometry around the Platinum atom is distorted square planer as N1-Pt1-N2 angle is 84.95 (3)° and Pt to its four co-ordinated atoms distances are [Pt-N = 2.071 (7) Å] and [Pt-Cl = 2.30 (5) Å]. The coordinated ligand atoms and Pt(II) are coplanar with r.m.s. deviation of 0.0119 Å. The planes of the two N(CH_3_)_2_ fragments are twisted at an angle of 12.53(1.05)°. The five membered ring formed by coordination of ligand atoms and metal is nonplaner with r.m.s. deviation of 0.1947 (4)Å and both the halogen atoms (Cl1 & Cl2) are displaced from the least square plane defined by (Pt1/N1/C3/C4/N2) by -0.2216 (1) Å & 0.0425 (1) Å respectively. In the crystal, C—H···Cl interactions link the molecules into (001) sheets (Table 2, Fig. 2).

### Experimental

In a round bottom flask took 50 ml of distilled water, 0.5 ml conc. HCL, 0.036 ml of tetramethylethylenediamine (tmeda) (0.24 mmol) and K_2_PtCl_4_ (0.24 mmol). Refluxed the mixture for about 2 hrs. The solution was changed to pale yellow, it was allowed to cool to room temperature then kept in refrigerator overnight. Pale yellow needles were formed which were filtered, washed with ethanol then with diethylether and dried on vacuum line.

### Refinement

All the C—H H-atoms were positioned with idealized geometry with C—H = 0.97 Å for methylene, & C—H = 0.96 Å for methyl groups. H-atoms were refined as riding with *U*_iso_(H) = k*U*_eq_(C, N), where k = 1.2 for methylene and k = 1.5 for methyl H-atoms.

### Crystal data

**Table:**

[PtCl_2_(C_6_H_16_N_2_)]	*F*(000) = 712
*M**_r_* = 382.20	*D*_x_ = 2.397 Mg m^−^^3^
Monoclinic, *I**a*	Cu *K*α radiation, λ = 1.54184 Å
Hall symbol: I -2ya	Cell parameters from 6609 reflections
*a* = 11.8893 (2) Å	θ = 4.0–74.3°
*b* = 6.0207 (1) Å	µ = 28.99 mm^−^^1^
*c* = 15.8036 (3) Å	*T* = 296 K
β = 110.549 (2)°	Cut needle, yellow
*V* = 1059.27 (3) Å^3^	0.22 × 0.21 × 0.07 mm
*Z* = 4	

### Data collection

**Table:**

Agilent SuperNova (Dual, Cu at zero, Atlas, CCD) diffractometer	2091 independent reflections
Radiation source: SuperNova (Cu) X-ray Source	2067 reflections with *I* > 2σ(*I*)
Mirror monochromator	*R*_int_ = 0.047
ω scans	θ_max_ = 74.5°, θ_min_ = 8.0°
Absorption correction: multi-scan (*CrysAlis PRO*; Agilent, 2012)	*h* = −14→14
*T*_min_ = 0.149, *T*_max_ = 1.000	*k* = −6→7
6899 measured reflections	*l* = −19→19

### Refinement

**Table:**

Refinement on *F*^2^	Hydrogen site location: inferred from neighbouring sites
Least-squares matrix: full	H-atom parameters constrained
*R*[*F*^2^ > 2σ(*F*^2^)] = 0.044	*w* = 1/[σ^2^(*F*_o_^2^) + (0.1007*P*)^2^] where *P* = (*F*_o_^2^ + 2*F*_c_^2^)/3
*wR*(*F*^2^) = 0.116	(Δ/σ)_max_ < 0.001
*S* = 1.03	Δρ_max_ = 1.83 e Å^−^^3^
2091 reflections	Δρ_min_ = −2.02 e Å^−^^3^
105 parameters	Extinction correction: *SHELXL97* (Sheldrick, 2008), Fc^*^=kFc[1+0.001xFc^2^λ^3^/sin(2θ)]^-1/4^
2 restraints	Extinction coefficient: 0.00105 (13)
Primary atom site location: structure-invariant direct methods	Absolute structure: Flack (1983), 1005 Friedel pairs
Secondary atom site location: difference Fourier map	Flack parameter: −0.02 (2)

### Special details

**Table:**

Geometry. All esds (except the esd in the dihedral angle between two l.s. planes) are estimated using the full covariance matrix. The cell esds are taken into account individually in the estimation of esds in distances, angles and torsion angles; correlations between esds in cell parameters are only used when they are defined by crystal symmetry. An approximate (isotropic) treatment of cell esds is used for estimating esds involving l.s. planes.
Refinement. Refinement of F^2^ against ALL reflections. The weighted R-factor wR and goodness of fit S are based on F^2^, conventional R-factors R are based on F, with F set to zero for negative F^2^. The threshold expression of F^2^ > 2sigma(F^2^) is used only for calculating R-factors(gt) etc. and is not relevant to the choice of reflections for refinement. R-factors based on F^2^ are statistically about twice as large as those based on F, and R- factors based on ALL data will be even larger.

### Fractional atomic coordinates and isotropic or equivalent isotropic displacement parameters (Å^2^)

**Table:**

	*x*	*y*	*z*	*U*_iso_*/*U*_eq_	
Pt1	0.5018 (3)	0.74348 (4)	0.3050 (2)	0.0267 (2)	
Cl1	0.4170 (2)	0.9830 (3)	0.37829 (16)	0.0534 (5)	
Cl2	0.3628 (2)	0.8443 (5)	0.16737 (16)	0.0595 (6)	
N1	0.6301 (5)	0.6421 (11)	0.4255 (3)	0.0315 (11)	
N2	0.5876 (5)	0.5269 (10)	0.2453 (3)	0.0354 (12)	
C1	0.5722 (6)	0.5088 (14)	0.4782 (4)	0.0471 (19)	
H1A	0.6327	0.4523	0.5318	0.071*	
H1B	0.5291	0.3870	0.4420	0.071*	
H1C	0.5174	0.6009	0.4949	0.071*	
C2	0.6976 (8)	0.828 (2)	0.4835 (5)	0.0472 (18)	
H2A	0.6438	0.9147	0.5034	0.071*	
H2B	0.7320	0.9203	0.4494	0.071*	
H2C	0.7604	0.7687	0.5351	0.071*	
C3	0.7209 (7)	0.5022 (14)	0.4027 (4)	0.0481 (18)	
H3A	0.7820	0.5970	0.3941	0.058*	
H3B	0.7599	0.4007	0.4519	0.058*	
C4	0.6581 (7)	0.3734 (14)	0.3176 (5)	0.0483 (16)	
H4A	0.7168	0.2957	0.2988	0.058*	
H4B	0.6051	0.2640	0.3288	0.058*	
C5	0.6643 (9)	0.6529 (18)	0.2053 (6)	0.058 (2)	
H5A	0.7253	0.7315	0.2522	0.087*	
H5B	0.6157	0.7570	0.1618	0.087*	
H5C	0.7014	0.5516	0.1762	0.087*	
C6	0.5055 (10)	0.3861 (19)	0.1728 (7)	0.073 (3)	
H6A	0.5513	0.2764	0.1549	0.109*	
H6B	0.4627	0.4771	0.1218	0.109*	
H6C	0.4493	0.3136	0.1948	0.109*	

### Atomic displacement parameters (Å^2^)

**Table:**

	*U*^11^	*U*^22^	*U*^33^	*U*^12^	*U*^13^	*U*^23^
Pt1	0.0225 (3)	0.0267 (3)	0.0262 (3)	−0.0006 (2)	0.00272 (17)	0.00193 (19)
Cl1	0.0403 (8)	0.0435 (11)	0.0769 (11)	0.0024 (7)	0.0214 (8)	−0.0180 (9)
Cl2	0.0491 (9)	0.0623 (17)	0.0450 (8)	0.0031 (10)	−0.0111 (6)	0.0197 (10)
N1	0.027 (2)	0.042 (4)	0.020 (2)	−0.002 (2)	0.001 (2)	0.002 (2)
N2	0.036 (2)	0.039 (3)	0.033 (2)	0.005 (2)	0.014 (2)	−0.001 (2)
C1	0.045 (4)	0.056 (5)	0.033 (3)	−0.009 (3)	0.005 (3)	0.013 (3)
C2	0.044 (4)	0.056 (6)	0.033 (4)	−0.025 (4)	0.003 (3)	−0.013 (4)
C3	0.038 (3)	0.061 (5)	0.036 (3)	0.019 (3)	0.002 (3)	0.003 (3)
C4	0.046 (4)	0.046 (4)	0.050 (4)	0.018 (3)	0.014 (3)	0.005 (3)
C5	0.068 (5)	0.064 (7)	0.057 (4)	0.005 (5)	0.040 (4)	0.010 (4)
C6	0.079 (6)	0.069 (7)	0.065 (5)	0.000 (5)	0.019 (4)	−0.037 (5)

### Geometric parameters (Å, º)

**Table:**

Pt1—N1	2.071 (7)	C2—H2B	0.9600
Pt1—N2	2.076 (6)	C2—H2C	0.9600
Pt1—Cl1	2.292 (4)	C3—C4	1.504 (11)
Pt1—Cl2	2.304 (5)	C3—H3A	0.9700
N1—C1	1.488 (7)	C3—H3B	0.9700
N1—C2	1.490 (10)	C4—H4A	0.9700
N1—C3	1.510 (8)	C4—H4B	0.9700
N2—C4	1.480 (9)	C5—H5A	0.9600
N2—C5	1.486 (9)	C5—H5B	0.9600
N2—C6	1.483 (10)	C5—H5C	0.9600
C1—H1A	0.9600	C6—H6A	0.9600
C1—H1B	0.9600	C6—H6B	0.9600
C1—H1C	0.9600	C6—H6C	0.9600
C2—H2A	0.9600		
			
N1—Pt1—N2	84.9 (3)	N1—C2—H2C	109.5
N1—Pt1—Cl1	91.9 (2)	H2A—C2—H2C	109.5
N2—Pt1—Cl1	176.7 (3)	H2B—C2—H2C	109.5
N1—Pt1—Cl2	177.2 (2)	C4—C3—N1	109.2 (5)
N2—Pt1—Cl2	92.3 (2)	C4—C3—H3A	109.8
Cl1—Pt1—Cl2	90.83 (16)	N1—C3—H3A	109.8
C1—N1—C2	108.4 (5)	C4—C3—H3B	109.8
C1—N1—C3	110.1 (6)	N1—C3—H3B	109.8
C2—N1—C3	106.9 (6)	H3A—C3—H3B	108.3
C1—N1—Pt1	109.7 (4)	N2—C4—C3	109.7 (6)
C2—N1—Pt1	114.1 (6)	N2—C4—H4A	109.7
C3—N1—Pt1	107.6 (3)	C3—C4—H4A	109.7
C4—N2—C5	112.4 (6)	N2—C4—H4B	109.7
C4—N2—C6	106.3 (7)	C3—C4—H4B	109.7
C5—N2—C6	107.4 (6)	H4A—C4—H4B	108.2
C4—N2—Pt1	106.0 (4)	N2—C5—H5A	109.5
C5—N2—Pt1	110.2 (5)	N2—C5—H5B	109.5
C6—N2—Pt1	114.5 (5)	H5A—C5—H5B	109.5
N1—C1—H1A	109.5	N2—C5—H5C	109.5
N1—C1—H1B	109.5	H5A—C5—H5C	109.5
H1A—C1—H1B	109.5	H5B—C5—H5C	109.5
N1—C1—H1C	109.5	N2—C6—H6A	109.5
H1A—C1—H1C	109.5	N2—C6—H6B	109.5
H1B—C1—H1C	109.5	H6A—C6—H6B	109.5
N1—C2—H2A	109.5	N2—C6—H6C	109.5
N1—C2—H2B	109.5	H6A—C6—H6C	109.5
H2A—C2—H2B	109.5	H6B—C6—H6C	109.5
			
N2—Pt1—N1—C1	−111.5 (5)	Cl1—Pt1—N2—C5	−82 (4)
Cl1—Pt1—N1—C1	69.7 (5)	Cl2—Pt1—N2—C5	77.3 (5)
Cl2—Pt1—N1—C1	−108 (5)	N1—Pt1—N2—C6	135.9 (7)
N2—Pt1—N1—C2	126.6 (4)	Cl1—Pt1—N2—C6	157 (4)
Cl1—Pt1—N1—C2	−52.2 (4)	Cl2—Pt1—N2—C6	−43.9 (7)
Cl2—Pt1—N1—C2	130 (5)	C1—N1—C3—C4	85.3 (7)
N2—Pt1—N1—C3	8.3 (5)	C2—N1—C3—C4	−157.2 (7)
Cl1—Pt1—N1—C3	−170.5 (5)	Pt1—N1—C3—C4	−34.3 (8)
Cl2—Pt1—N1—C3	11 (5)	C5—N2—C4—C3	76.8 (8)
N1—Pt1—N2—C4	19.1 (5)	C6—N2—C4—C3	−165.9 (6)
Cl1—Pt1—N2—C4	40 (4)	Pt1—N2—C4—C3	−43.7 (7)
Cl2—Pt1—N2—C4	−160.7 (5)	N1—C3—C4—N2	53.2 (8)
N1—Pt1—N2—C5	−102.8 (5)		

### Hydrogen-bond geometry (Å, º)

**Table:**

*D*—H···*A*	*D*—H	H···*A*	*D*···*A*	*D*—H···*A*
C1—H1*B*···Cl1^i^	0.96	2.79	3.724 (8)	166
C4—H4*A*···Cl1^ii^	0.97	2.82	3.596 (7)	137

Symmetry codes: (i) *x*, *y*−1, *z*; (ii) *x*+1/2, −*y*+1, *z*.
